# Smoking reduction using an electronic nicotine delivery system (ENDS) with nicotine delivery similar to combustible cigarettes

**DOI:** 10.1186/s12954-024-01064-0

**Published:** 2024-07-29

**Authors:** Jed E. Rose, Frederique M. Behm, Gal Cohen, Perry N. Willette, Tanaia L. Botts, David R. Botts

**Affiliations:** Rose Research Center, LLC, 7240 ACC Blvd, Raleigh, NC, 27617 USA

**Keywords:** Nicotine, Cigarette smoking, Electronic cigarette, Dependence

## Abstract

**Background:**

Electronic nicotine delivery systems (ENDS) offer a promising approach to tobacco harm reduction, but many people use both ENDS and combustible cigarettes (“dual use”), which undermines potential risk reduction. To explore the role of ENDS nicotine delivery in promoting switching to ENDS, we conducted a study in which people who smoked cigarettes were offered an ENDS that had previously been shown to replicate the rapid nicotine pharmacokinetics of combustible cigarettes (BIDI^®^ Stick).

**Methods:**

Twenty-five cigarette smoking adults, not seeking smoking cessation treatment, but open to using ENDS as a cigarette substitute, were provided with a 12-week supply of BIDI^®^ Stick in tobacco or menthol flavors, during a study that included seven biweekly sessions and a 6-month follow-up. Daily diaries assessed ENDS and cigarette use, and exhaled carbon monoxide (eCO) served as an objective marker of smoke intake. Subjective ratings were collected to assess the rewarding properties of ENDS and combustible cigarettes, and indices of nicotine dependence.

**Results:**

Over 12 weeks, ENDS use increased to an average of 15.8 occasions per day (*SD* = 20.2) and self-reported cigarette consumption decreased by 82% from 16.7 cigarettes/day (*SD* = 6.0) at baseline to 3.0 cigarettes/day (*SD* = 4.1) at week 12. The eCO level decreased by 27% from an average of 20.0 ppm (*SD* = 9.8) at baseline to 14.5 ppm (*SD* = 9.9) at week 12. Four of 25 participants completely switched to ENDS and were smoking abstinent during weeks 9–12. At 6 months one participant was confirmed to be abstinent. Ratings of subjective reward for the ENDS were very similar to those of participants’ usual brands of cigarettes. Dependence level was lower for the ENDS than for combustible cigarettes.

**Conclusions:**

In this study, the ENDS effectively replicated the subjective rewarding effects of participants’ usual brands of cigarettes and led to a substantial reduction in reported cigarettes/day. Exhaled CO showed less of a decrease, possibly due to compensatory smoking behavior and/or the timing of eCO measurements that might not have reflected smoke intake throughout the day. The relatively low rate of sustained smoking abstinence at 6 months suggests that additional approaches continue to be needed for achieving higher rates of complete switching.

**Trial registration:**

ClinicalTrials.gov identifier NCT05855343.

**Supplementary Information:**

The online version contains supplementary material available at 10.1186/s12954-024-01064-0.

## Introduction

Tobacco harm reduction is an important public health strategy that refers to substituting lower-risk nicotine and tobacco products in place of high-risk products, for individuals who are not able to or do not desire to relinquish nicotine [1–3]. In recent years, electronic nicotine delivery systems (“ENDS”), or “e-cigarettes,” have gained popularity as a tobacco harm reduction strategy, to help individuals who smoke cigarettes quit smoking or substantially reduce their consumption of cigarettes. Evidence from randomized controlled trials and real-world studies [4, 5] support superior efficacy of ENDS in smoking cessation treatment as compared with previous forms of nicotine replacement therapy (NRT). Furthermore, the US FDA has authorized marketing of several ENDS products as “appropriate for the protection of public health” [6]. While concerns remain about dependence potential for adolescents, the use of ENDS for adults seeking to reduce or eliminate combustible cigarette use has gained increasing support [7].

A key challenge with ENDS use is that there is a low rate of complete switching from combustible cigarette use [4, 8, 9]. Generally, these studies of switching behavior do not select highly motivated treatment seekers and find that only 5–15% completely relinquish combustible cigarettes and adopt exclusive ENDS use. While smoking reduction is consistent with the goal of tobacco harm reduction, a low rate of switching results in individuals continuing to be exposed to the toxins from cigarette smoke. The finding of low switching rates raises the question of whether there are critical factors missing from current ENDS that render them less effective in fully replacing cigarettes. One factor could be an inadequate pharmacokinetic (PK) profile, e.g., rate of nicotine delivery or overall dose. For example, Foulds et al. [8] found that a 36 mg/mL nicotine concentration in the e-liquid led to more days of smoking abstinence than lower concentrations (0 mg/mL or 8 mg/mL), and Goldenson et al. [10] reported that e-liquid nicotine concentrations > 20 mg/mL were associated with higher switching rates. Hajek et al. [11] tested several ENDS brands and found they did not deliver nicotine as efficiently as combustible cigarettes. ENDS that use e-liquids having alkaline pH typically deliver nicotine less efficiently to the lung and brain than conventional cigarettes [12]. ENDS using nicotine salt formulations, which are more efficient at delivering nicotine, nonetheless often fall short of providing the same peak nicotine levels obtained by smoking [13]. Recently, one particular ENDS device (BIDI^®^ Stick), which uses a nicotine salt formulation, was reported to fully match the plasma nicotine levels of cigarette smoking [14]. This ENDS offers a tool to evaluate the importance of nicotine pharmacokinetics in explaining the observed switching rates from combustible cigarettes to ENDS. If an insufficient nicotine delivery were the main reason for low switching rates, then this ENDS should yield a relatively high switching rate.

## Methods

This open-label pilot study was conducted as an initial evaluation of the behavioral and subjective responses to use of an ENDS with similar pharmacokinetic profile as combustible cigarettes, with the objective of promoting switching from cigarettes to ENDS. The study used as a comparison the historical database from other studies, including ones from our research center, which followed similar methods, using a variety of marketed ENDS products.

### Design

This study was an open-label clinical trial that evaluated 25 adults who smoked and who were *not* seeking smoking cessation treatment, but who expressed interest in switching from combustible cigarettes to ENDS. Participants were recruited from the Raleigh and Charlotte metropolitan areas of North Carolina, using online advertisements. Eligibility criteria included being between the ages of 22–65, smoking at least 10 combustible cigarettes per day for the last 12 months, and having an eCO reading of at least 10 ppm. Exclusion criteria were use or planned use of any smoking cessation treatment during the study, current use of alternative nicotine products (including ENDS) or illicit drugs, high blood pressure (systolic > 150 mm Hg, diastolic > 95 mm Hg), body mass index (BMI) < 15.0 kg/m^2^ or > 40 kg/m^2^, serious medical or psychiatric disease, pregnancy or nursing. Informed consent was obtained from all participants; the study was reviewed and approved by the Advarra Institutional Review Board and registered with ClinicalTrials.gov (NCT05855343). Participants were compensated for participation ($50 for completing Study Visit 1; $600 total for completing Study Visits 2 through 7; $5/day for responding to daily surveys, totaling $420 for 84 days; and $75 for a 6-month follow-up visit). The BIDI^®^ Sticks were provided free of charge, and were dispensed at the visits in amounts sufficient to last until the next visit, according to each participant’s baseline smoking rate. One BIDI^®^ Stick contained 82.6 mg nicotine, which was approximately equivalent in nicotine delivery to 40 cigarettes (2 packs), based on measures of cigarette nicotine yield by Hammond et al. [15] during human smoking (2 mg/cigarette),. The number of BIDI^®^ Sticks dispensed to each participant was equal to half of their baseline number of packs of cigarettes smoked per day, along with an extra supply of 25% to prevent running out. For example, if someone smoked 20 cigarettes/day at baseline, then over a 14-day period they would receive 1.25 × 20 × 14/40 (rounded up to the nearest whole number) = 9 BIDI^®^ Sticks.

After completing a medical screening session, participants were scheduled for their first study session, in which they sampled two flavors of BIDI^®^ Stick, “Arctic Menthol” and “Classic Tobacco Leaf,” in order to ascertain their preference (they were allowed to change their preference over the product use period if desired). They received a 1-week supply of their preferred ENDS and the instruction to switch completely from their own brand of cigarette to ENDS within the week. Sessions 2 through 7 occurred at 2, 4, 6, 8, 10 and 12 weeks after the first study session, with participants receiving their preferred flavored ENDS sufficient to last through the next session. Participants were instructed to use ENDS prior to smoking any combustible cigarettes, as we had previous evidence this might devalue cigarettes and facilitate switching ( [16], see Additional file 1).

### Sample size

In a prior research study measuring eCO reductions with use of ENDS [9], we observed a reduction of eCO by approximately 40% over 8 weeks. Assuming the ENDS used in this study would yield a greater reduction due to its higher nicotine delivery, we calculated that a sample size of 25 had a power of 97% to detect a decrease in eCO of 50% from baseline to week 12, using alpha (2-tailed) = 0.05.

### BIDI^®^ stick

This ENDS was a marketed, self-contained, breath-actuated, disposable electronic cigarette, which contained 1.4 mL of 6% nicotine (59 mg/mL), benzoic acid, propylene glycol, vegetable glycerol and flavoring. ENDS supplies were purchased from BIDI Vapor, LLC (Melbourne, FL). The prior study by Fearon et al. [14] showed that the peak plasma nicotine concentrations attained after controlled use of the BIDI^®^ Stick matched the average nicotine yield of participants’ usual brands of cigarettes (15–18 ng/mL) and after *ad libitum* use tended to exceed those of the usual brand. Additionally, subjective ratings of rewarding effects were similar to the usual brand, although not quite as high for psychological reward and perceived sufficiency of nicotine delivery.

### Dependent measures

Cigarette and ENDS use were monitored using daily diaries collected via *eResearch*, a proprietary mobile electronic application for clinical research studies downloaded by participants from iOS or Android app stores. Daily notifications were pushed to participants to collect self-reported number of cigarettes smoked daily, the number of ENDS use occasions and average number of ENDS puffs per occasion.

At each session, eCO was measured using a Vitalograph monitor (model 2900) as an objective measure of smoking reduction and smoking abstinence. Sessions were generally conducted between 9AM and 3PM. At the 6-month follow-up, only participants claiming to be abstinent from smoking were asked to come in for an eCO measurement.

Additional measures collected at each session were ratings of the rewarding and aversive effects of product use, assessed by the previously validated modified Cigarette Evaluation Questionnaire (mCEQ) [17]. This questionnaire had scales for satisfaction (“satisfying,” “tastes good,” “enjoy smoking”), psychological reward (“calm you down,” “feel more awake,” “feel less irritable,” “helps you concentrate,” “reduces hunger”), aversion (“makes you dizzy,” “makes you nauseated”), “enjoy the sensations in the throat and chest” (single-item assessment), and “reduce your craving” (single-item assessment), using 7-point rating scales, ranging from “not at all” to “extremely”. These items were assessed for the first cigarette smoked or ENDS used for each day.

The level of cigarette dependence was assessed at baseline, week 2 and week 12 using the Fagerström Test for Nicotine Dependence (FTND) [18]. To evaluate the level of dependence on the ENDS product compared to combustible cigarettes at the 6-month follow-up, the 4-item Patient-Reported Outcomes Measurement Information System (PROMIS) dependence questionnaire [19] was administered. Two versions were used, one for each product, and the ENDS version specifically referred to the BIDI^®^ Stick in each question. The four items in the questionnaire assessed the degree of “intolerable craving” when unable to use the product, using the product “without thinking,” “dropping everything” to obtain the product, and using the product more before entering a situation in which it “is not allowed.” A total score was calculated by summing the item scores, which used a 1–5 scale (ranging from “never” to “always”).

### Data analysis

Because of the exploratory nature of this study, which did not have a formal control group, descriptive statistics were tabulated for the dependent measures. A limited number of *post hoc* statistical tests were conducted, as well as comparisons between study products, to examine behavioral change during the study.

The definition of 4-week continuous smoking abstinence was a self-report of no smoking between weeks 9–12 confirmed by eCO readings < 5 ppm [20]. Point abstinence at 6 months was defined as an eCO-confirmed report of smoking abstinence for a 7-day period.

## Results

### Participants

Twenty-five participants (three Hispanic or Latino, 11 white, 10 Black, four others; 15 males, 10 females) were enrolled. They smoked on average 16.9 cigarettes/day (*SD* = 5.4), with five smoking non-menthol brands and 20 smoking menthol brands. Their mean age was 50.4 years (*SD* = 9.8) and they had been smoking regularly for 29.5 years (*SD* = 9.1). At baseline (screening) the mean FTND score was 5.9 (*SD* = 2.0), and baseline eCO level was 19.3 ppm (*SD* = 9.3).

### Cigarette and ENDS use

**Fig. 1:**
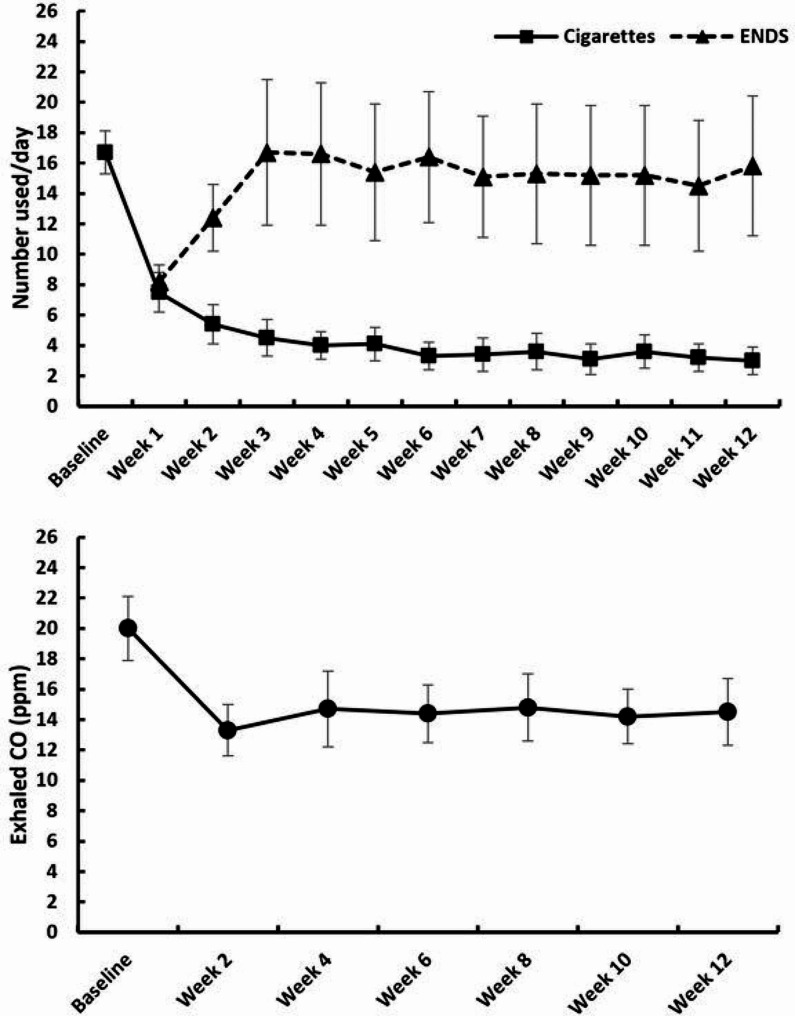
Upper panel: Weekly mean (± s.e.m.) numbers of cigarettes smoked/day and ENDS uses/day. Lower panel: Exhaled CO readings (mean ± s.e.m.) at each session

As shown in Fig. 1, cigarette use declined markedly over the 12 weeks of ENDS use, from 16.7 cigarettes/day (*SD* = 6.0) at baseline to 3.0 cigarettes/day (*SD* = 4.1) at week 12 (*n* = 19 complete datasets); *F*(12,216) = 27.66, *p* < .0001 for the effect of time. Concurrently, ENDS use increased over time from 8.2 occasions/day (*SD* = 4.7) at week 1 to 15.8 at week 12 (*SD* = 20.2); *F*(11,198) = 2.21, *p* = .02. The average number of puffs per occasion did not change over time (mean = 7.9, *SD* = 7.4). Exhaled CO decreased in the first 2 weeks and leveled off subsequently; *F*(6, 120) = 3.11, *p* = .007 for the effect of time, from an average of 20.0 ppm (*SD* = 9.8) at baseline to 14.5 ppm (*SD* = 9.9) at week 12, a decrease of 27.4%.

Over the 12 weeks, the number of self-reported daily ENDS use occasions was negatively correlated with cigarettes/day (*r*(24)=-0.48, *p* = .02,excluding one outlier with a value of 79 occasions/day as compared with the mean of 11.1 (*SD* = 6.0)).

At the 6-month follow-up, self-reported cigarette use remained lower than baseline values: 11.1 cigarettes/day (*SD* = 4.4) at 6 months vs. 17.4 cigarettes/day (*SD* = 6.0) at baseline (*n* = 16 participants assessed at both timepoints); *F*(1,15) = 13.77, *p* = .002. In contrast, eCO did not differ: 18.7 ppm (*SD* = 7.3) at baseline vs. 18.9 ppm (*SD* = 12.3) at 6 months (*F*(1,17) = 0.01, *p* = .9, *n* = 18). Mean number of self-reported ENDS use occasions/day was 10.1 (*SD* = 10.1), based on *n* = 10 respondents. Also, for those reporting both ENDS and cigarette use, the prior negative correlation between ENDS use occasions and cigarettes/day was no longer apparent (*r*(10) = 0.47, *p* = .17).

Complete 4-week continuous abstinence from combustible cigarettes at week 12 was 16% (4/25); 90%CI [6, 33]. Point (7-day) abstinence at 6 months was 4% (1/25), 90%CI [0, 18].

### Subjective ratings

**Fig. 2:**
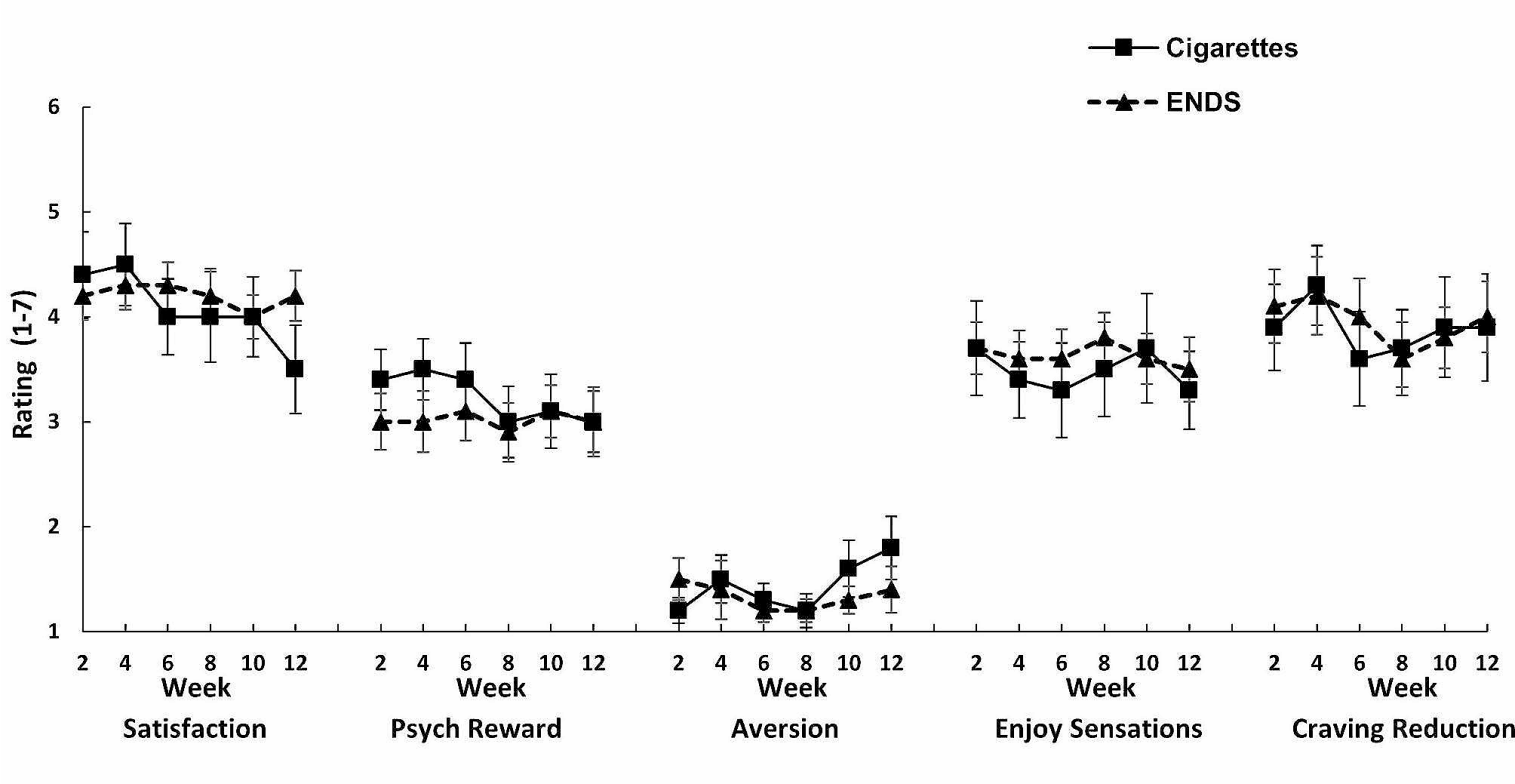
Ratings of the rewarding/aversive effects of conventional cigarettes and ENDS, assessed at sessions conducted over the study period. Points represent the means (± s.e.m.)

Figure 2 presents the subjective ratings of cigarettes and ENDS over the course of the study. Ratings for all reward scales were very similar for the two products.

The level of dependence on cigarettes, as assessed by the FTND questionnaire at baseline, week 2 and week 12, decreased over time: 6.6 (*SD* = 1.5), 4.7 (*SD* = 1.8), and 4.1 (*SD* = 1.4), respectively; *F*(2,28) = 18.93, *p* < .0001 (*n* = 15). At 6 months, for those reporting smoking (*n* = 16), the mean FTND score remained lower than baseline: 5.1 (*SD* = 2.4) vs. 6.1 (2.1) (*F*(1,15) = 5.32, *p* = .04). At all time points, 87–93% of respondents reported smoking their first cigarette within 60 min of awakening. At 6 months, the PROMIS dependence score was lower for ENDS than combustible cigarettes, based on those who reported using both products in the last seven days (paired *t*(7) = 3.0, *p* = .02; mean score = 8.3(*SD* = 2.8) vs. 12.9(*SD* = 2.5).

## Discussion

The results of this study suggest that providing an ENDS nicotine delivery comparable to that of cigarettes led to a substantial reduction in smoking behavior. Self-reported cigarette consumption showed a substantial decrease of 82% and this reduction was correlated with the number of ENDS use occasions during the first 12 weeks of the study, as was reported in previous studies using different ENDS devices [9, 21]. Moreover, eCO levels decreased by 27.4% at 12 weeks. Reductions in smoking of this magnitude are likely to be clinically meaningful in that a reduction of 20–50% in self-reported cigarettes smoked per day has been associated with reductions in risk of smoking-related cancers [22].

Nonetheless, use of an ENDS product with nicotine delivery similar to combustible cigarettes did not yield a markedly higher smoking abstinence rate than seen in studies using different ENDS products with lower nicotine yield. In particular, the rates of smoking abstinence at 12 weeks and 6 months, based on CO-verified abstinence outcomes, of 16% and 4%, were not demonstrably higher than the typical 5–15% reported in studies using other ENDS devices [4, 8, 9]. While prior work has shown that delivering higher doses of nicotine yields higher abstinence rates [8, 10], matching the nicotine yield of a cigarette may not be sufficient in itself to ensure high switching rates.

There was a discrepancy between eCO and cigarettes/day as indices of smoking reduction, which could have been due to several factors, including: (1) under reporting of actual cigarette consumption. For example, three of seven participants who reported smoking no cigarettes during week 12 had eCO levels clearly indicative of smoking (four of seven had been confirmed to be abstinent). However, it seems unlikely that underreporting would account for the entire discrepancy between cigarettes/day and eCO; (2) compensatory smoking behavior, i.e., smoking fewer cigarettes more intensively. This explanation seems less likely, given that participants had access to an ENDS that provided ample nicotine; and/or (3) the timing of eCO assessments, which typically occurred in the middle of the day, and therefore may not have reflected the cumulative smoke intake throughout the entire day. The time to the first cigarette, collected in the FTND questionnaire, showed that most participants started smoking within 60 min of awakening, despite the reduction in total number of cigarettes/day. Thus, the eCO measurement may have been biased relative to what would have occurred had eCO been measured later in the day. A similar discrepancy between self-reported cigarettes/day and eCO has been noted in other studies [21, 23]. Notwithstanding this discrepancy, the rate of complete smoking abstinence, which took both measures into account, was 16% at 12 weeks and 4% at 6 months, no higher than expected with other ENDS products.

In considering explanations for why the ENDS fell short of achieving a higher rate of complete switching, several possibilities merit consideration. First, might the ENDS not have been as rewarding or satisfying as cigarettes, due to components missing from ENDS that are present in cigarette smoke? However, the subjective ratings of the two products were highly comparable, replicating over a period of weeks the main results of the Fearon et al. [14] laboratory study. While not ruling out the possibility that missing constituents (e.g., monoamine oxidase inhibitors, minor tobacco alkaloids [24]) play a role, these results argue against that explanation.

A second possibility is that the habit of smoking is deeply entrenched and triggered by a wide range of conditioned cues in the individual’s environment. Thus, even if a product matches the reinforcing qualities of a cigarette, it will not necessarily displace the use of a behavior that has been consistently reinforced for many years (the study cohort had been smoking, on average, for three decades). Behavioral theories of choice [25] would predict that equally reinforcing outcomes with a similar history of reinforcement would yield a 50% preference for each outcome rather than exclusive preference for either outcome, in the absence of other overriding influences. These influences could include psychosocial factors that might impede or facilitate switching behavior. For example, there was no clear statement provided to participants that ENDS are less harmful than smoking combustible cigarettes. If such a conclusion were endorsed by regulatory authorities it would no doubt provide an additional motivational influence that could affect switching outcomes. Indeed, there is evidence that among people who smoke, those who believe ENDS to be as harmful as combustible cigarettes have a significantly lower rate of switching [26].

Finally, the continued bolus nicotine delivery from ENDS may sustain nicotine dependence processes through reinforcement or other neuropharmacological adaptations in the brain, similar to what a cigarette does. Among those using both ENDS and cigarettes, the levels of dependence on combustible cigarettes and ENDS at 6 months, reflected by the PROMIS questionnaire, showed continued dependence on ENDS, albeit less than for cigarettes. This result is in accord with other studies that found lower dependence scores on ENDS than cigarettes in a group of individuals using both products [27, 28]. Nonetheless, maintaining nicotine dependence with ENDS use may undermine efforts to completely quit smoking unless specific interventions also address breaking the dependence on combustible cigarettes. For example, using medications such as varenicline in combination with ENDS appears to yield substantially higher switching outcomes [21, 29]. In another study, using a novel drug combination, the ratings of reward for combustible cigarettes were found to be lower than those of ENDS [30], which may have enhanced switching. Thus, it may be helpful not only to match the rewarding aspects of smoking but to have greater reinforcement for ENDS to overcome the long-standing addictive behavior of smoking.

While this study offered insights into the role of factors influencing switching behavior, it had several limitations. One limitation was that the nicotine levels and rate of nicotine delivery were not measured in the study participants, and we are relying on previous published results to infer that the BIDI^®^ Sticks matched the pharmacokinetics of combustible cigarettes. An additional limitation was the absence of objective biomarkers of smoke intake other than eCO, such as urinary NNAL (4-(methylnitrosamino)-1-(3-pyridyl)-1-butanol), or urinary cyanoethyl mercapturic acid (2CyEMA) [31, 32]. Additionally, measures of propylene glycol in urine or plasma can be used as a biomarker of ENDS use [33], although the sensitivity and specificity of the measure will be diminished when the e-liquid has a high nicotine concentration (low propylene glycol:nicotine ratio). Furthermore, smoking topography measures and/or multiple eCO measurements throughout the day could help resolve the discrepancy between eCO and self-reported cigarettes/day. The study was also limited by the small number of people studied, which places fairly wide limits on the confidence intervals around the smoking abstinence rates and potentially limits the generalizability of the findings. Nonetheless, if the essential factors for complete switching were rapid nicotine delivery and providing a cigarette-like rewarding experience, confirmed by subjective ratings, then the percentage of participants who achieved smoking abstinence should arguably have been greater than 15% at 12 weeks and 4% at 6 months.

In summary, the current study suggests that providing a rewarding alternative to combustible cigarettes in the form of an ENDS with comparable nicotine yield may lead to a marked reduction in smoking behavior and a modest rate of complete switching. The substantial reduction in cigarettes/day offers encouragement that such an approach can be enhanced to reach the ultimate goal of assisting cigarette dependent individuals to relinquish a harmful behavior.

### Electronic supplementary material

Below is the link to the electronic supplementary material.

Supplementary Material 1

## Data Availability

The datasets used and/or analyzed during the current study are available from the corresponding author on reasonable request.
