# Supplementary material for: Smoking reduction using an electronic nicotine delivery system (ENDS) with nicotine delivery similar to combustible cigarettes
Source: Harm Reduct J. 2024 Jul 29;21:142. doi: 10.1186/s12954-024-01064-0 (PMC11285397; doi:10.1186/s12954-024-01064-0)
Supplement: Supplementary file 1 — Supplementary Material 1 [file 12954_2024_1064_MOESM1_ESM.pdf]

A CIGARETTE DEVALUATION STRATEGY TO FACILITATE SWITCHING AWAY FROM COMBUSTIBLE CIGARETTES

Gal Cohen, Tanaia Botts, David Botts, Perry Willette, Jed E. Rose. Rose Research Center, NC

SRNT 2024
EDINBURGH, SCOTLAND
MARCH 20-23

Rose Research Center, LLC

Summary

Introduction. Behavioral supportive approaches have been shown to increase odds of stopping smoking. This study explored a strategy for aiding adults who smoke to switch away from combustible cigarettes (CC) to an electronic nicotine delivery system (ENDS), whereby participants were instructed to use an ENDS immediately prior to smoking any CC. By using ENDS before CC to achieve pre-emptive nicotine satiation, the aim was to reduce CC use by behaviorally devaluing cigarettes.

Objectives. This behavioral approach was designed to devalue cigarettes in a targeted manner for people who smoke, while not increasing the attractiveness of ENDS products and risk of nicotine use initiation for people who do not smoke. This approach may also have utility for NRT products to increase odds of cessation.

Methods. A pilot study compared cigarette craving and satisfaction ratings of six smokers when CCs were smoked either right after use of an ENDS (Mark Ten 4.0%) or after a period of abstinence, in a counterbalanced design. Participants continued using e-cigarettes for a 1 week period, with instructions to use ENDS immediately prior to any CC smoked.

A subsequent field study enrolled 50 participants in a 12-week switching protocol with similar instruction to use ENDS (JUUL 5.0%) immediately prior to any CC smoked. Expired air carbon monoxide (CO) was measured as an objective marker of CC use and abstinence. Daily self-reports of CC and ENDS use, craving, satisfaction, and adherence to the instruction were also collected.

ANOVA and logistic regression were used to relate abstinence to adherence, while controlling for other individual subject variables. A threshold of 80% daily self-reported cumulative adherence was used.

Results. The pilot study suggested that CCs were rendered less satisfying when smoked immediately after an ENDS. In the field study, participants who were adherent to the instruction (8/50) showed lower smoking satisfaction, reduced CC craving and CO, as well as a higher rate of smoking abstinence at weeks 9-12 (OR=7.0, 90% CI=1.5-33.2, P=0.02).

Conclusions. Cigarette devaluation may be a simple, useful and targeted approach to help people who smoke successfully switch away from CCs to an ENDS.

Limitations. Follow-on randomized controlled trials are needed to evaluate the efficacy of this approach.

Demographics (baseline)

		Study 1	Study 2
**Sample size**	n	**n=6**	**n=50**
**Black**	N (%)	**3 (50%)**	**13 (26%)**
**Hispanic**	N (%)	**0 (0%)**	**5 (10%)**
**Female**	N (%)	**5 (83%)**	**23 (46%)**
**Age**	mean (SD)	**42.0 (11.1)**	**46.6 (10.7)**
**CPD**	mean (SD)	**9.3 (2.3)**	**19.5 (6.9)**
**FTND**	mean (SD)	**4.7 (2.3)**	**4.2 (1.5)**
**Exhaled CO (ppm)**	mean (SD)		**24.0 (10.4)**

This research was funded by a grant from the Foundation for a Smoke-Free World ("FSFW"), a US nonprofit 501(c)(3) private foundation. FSFW had no role in the planning or execution of this study, data analysis, or publication of results.

	Tobacco Industry	E-cigarette and Nicotine Product Industry	Pharma Industry
The work being presented has received funding or other means of support from any of the following sources:	**NO**	**NO**	**NO**
Any of the authors have received funding (including consultancy) from any of the following sources in the past 5 years:	**YES**	**YES**	**YES**

Cigarette Devaluation Approach

When urge to smoke cigarette arises → **Use ENDS immediately prior to any use of cigarette**

Study 1: Acute use followed by 1-week ambulatory study (n=6)

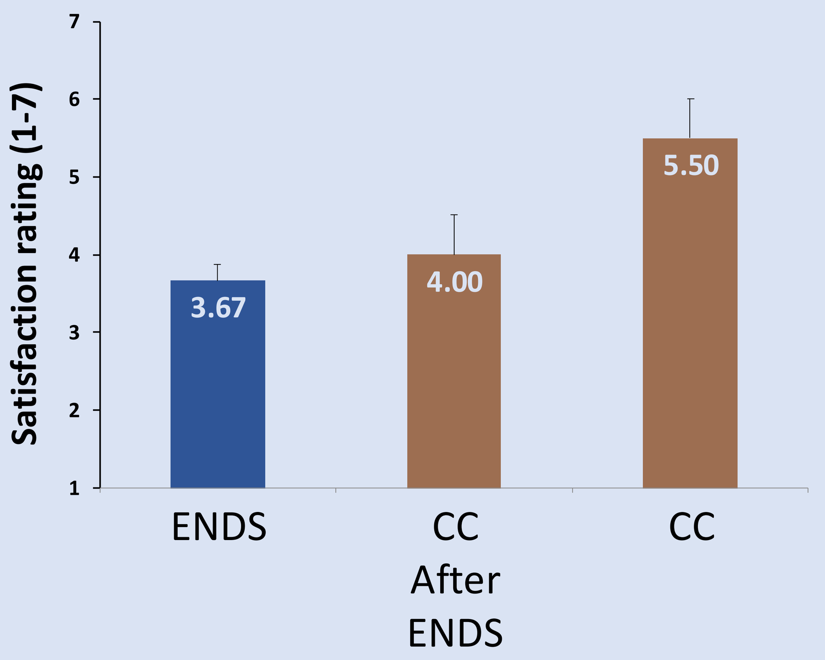

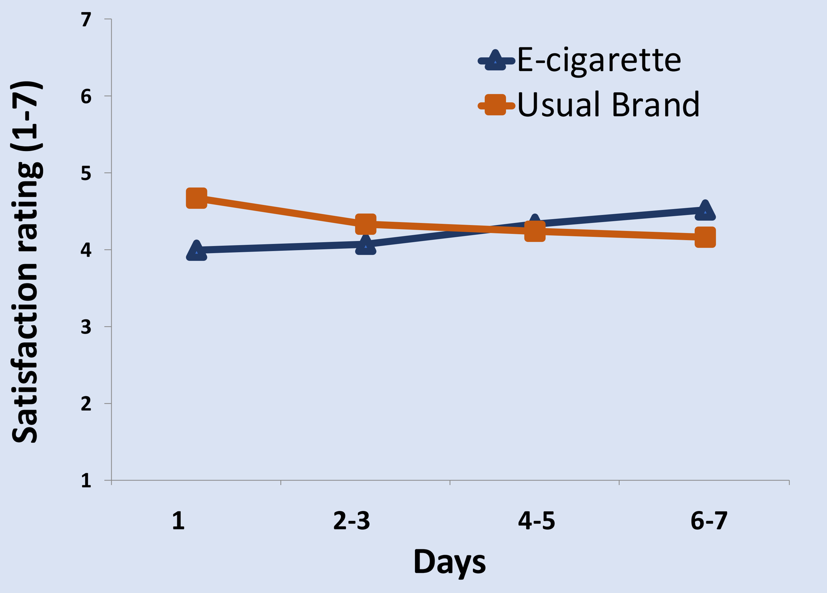

Study 2: 12-week ambulatory switching study (n=50)

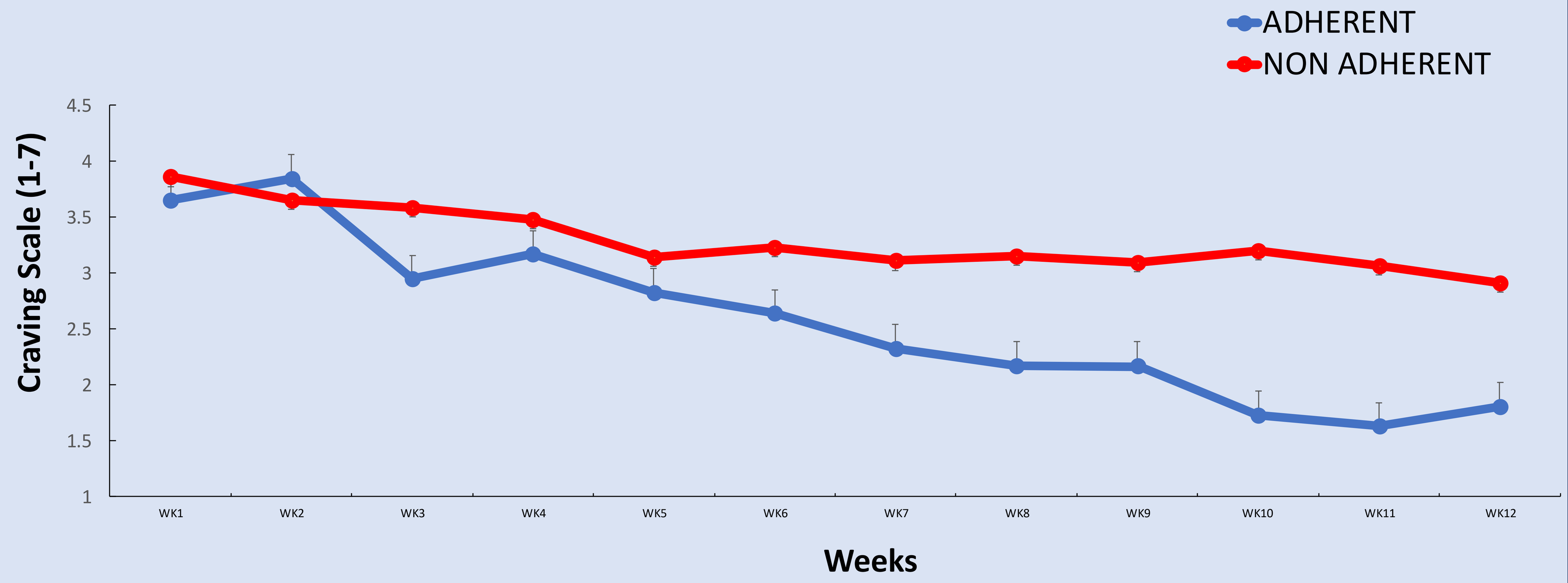

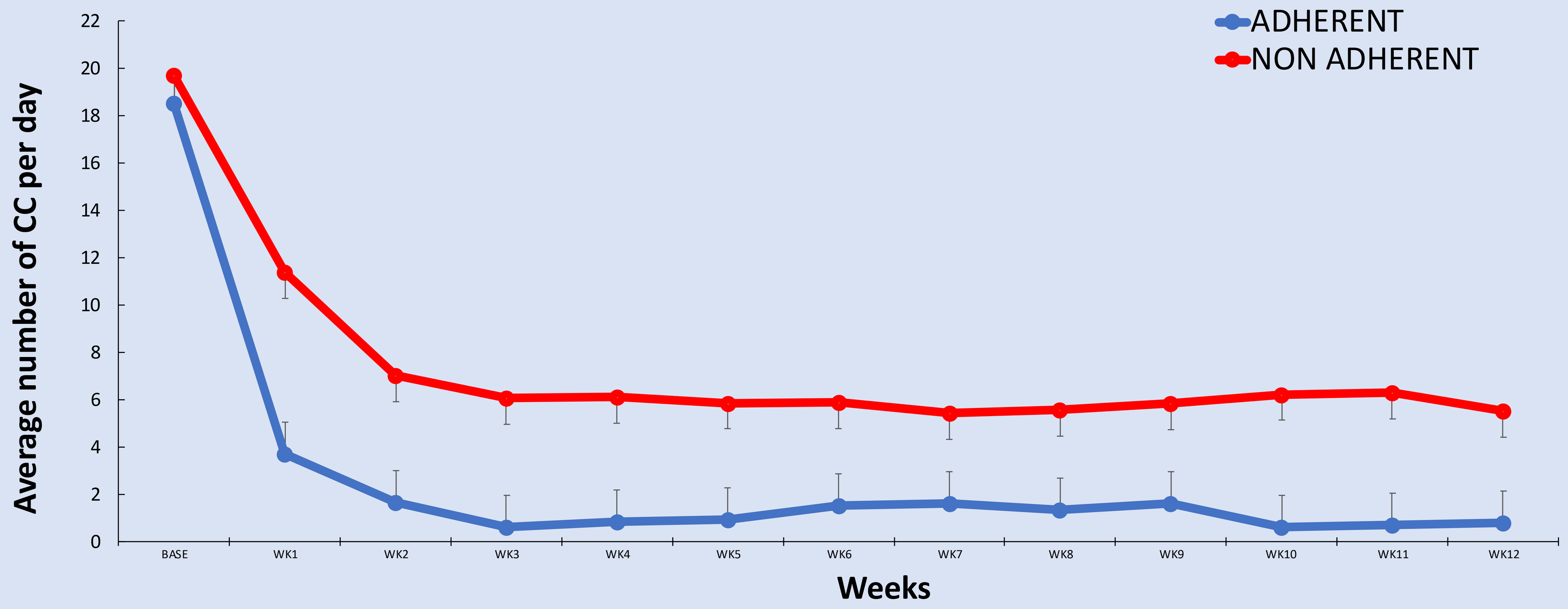

Conclusions

- **Use of ENDS, and in particular before use of cigarettes, was associated with reduction in satisfaction with cigarettes.**
- **Adherence to a simple cigarette devaluation approach was associated with a greater reduction in cravings and CPD.**
- **Extinguishing cravings occurs on a longer timescale than reduction in smoking, showing the importance of adherence.**
- **These finding may also have relevance for increasing the efficacy of other stop-smoking products including medicinal NRTs and nicotine substitution products.**
